# Supplementary figures and images for: Effect of the flavonoid baicalin on the proliferative capacity of bovine mammary cells and their ability to regulate oxidative stress
Source: PeerJ. 2019 Mar 5;7:e6565. doi: 10.7717/peerj.6565 (PMC6407502; doi:10.7717/peerj.6565)

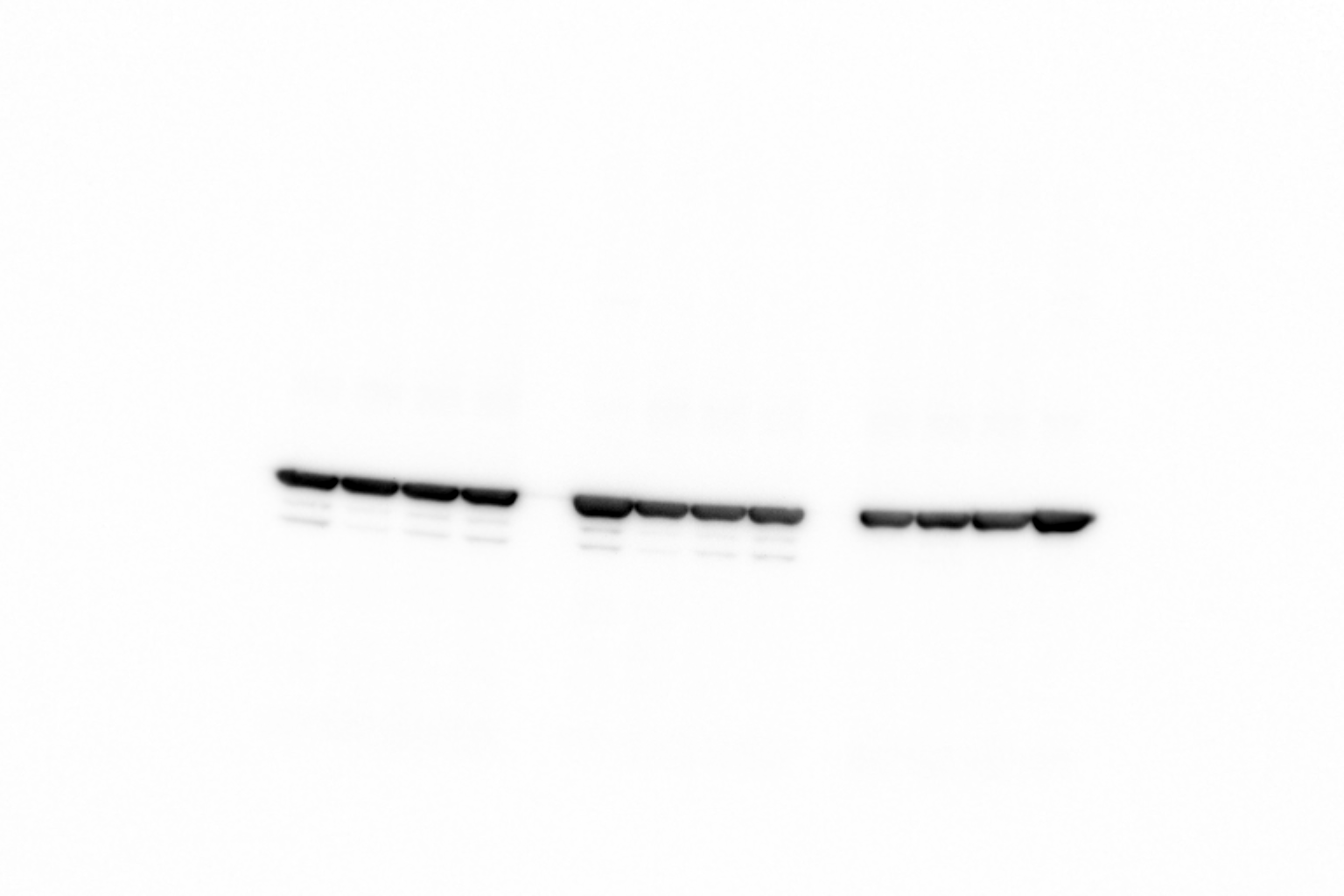

Supplement: Supplemental Information 1 — Actine, Catalase, Nrf-2. [file peerj-07-6565-s001.zip › actine.tif]

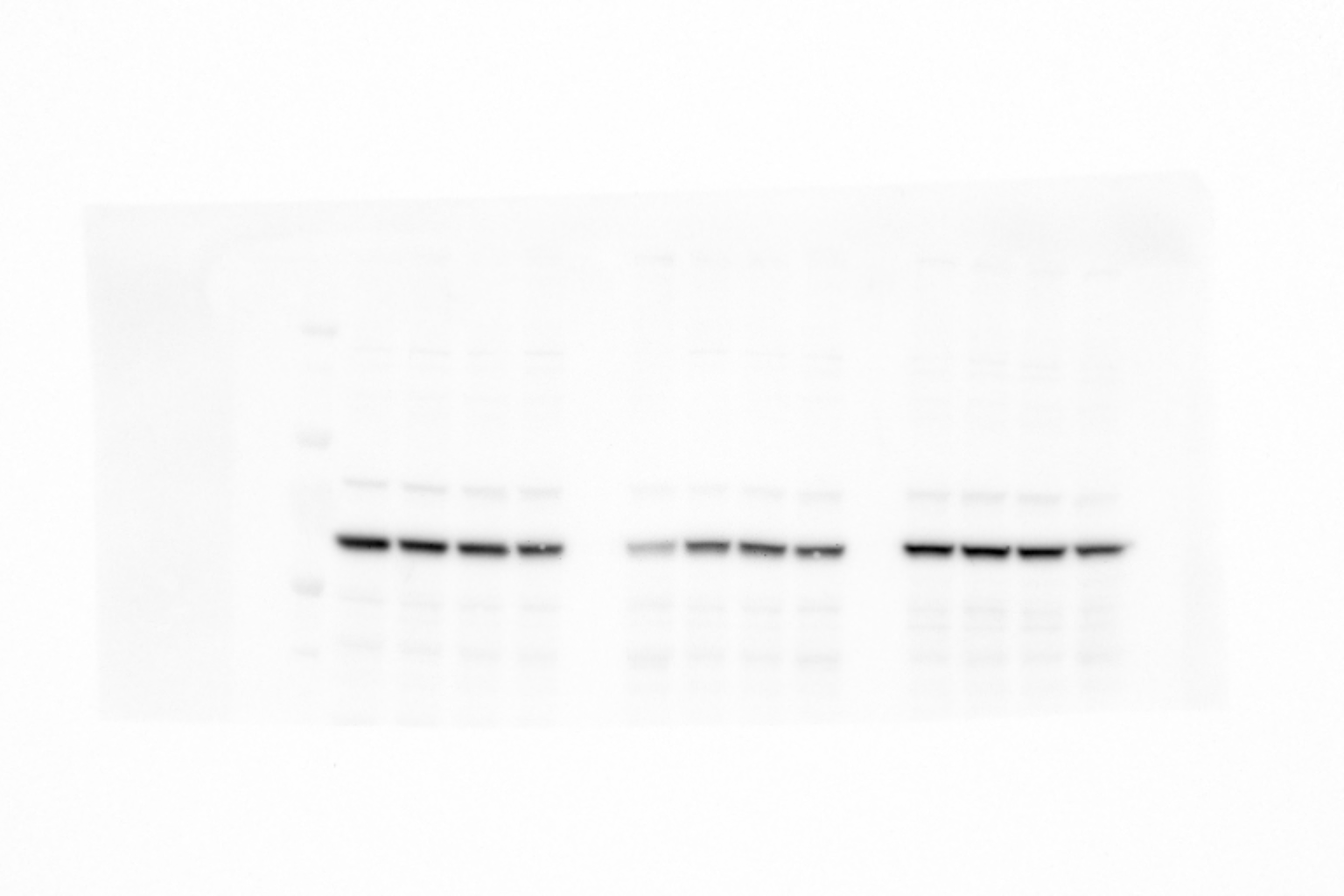

Supplement: Supplemental Information 1 — Actine, Catalase, Nrf-2. [file peerj-07-6565-s001.zip › catalase-high-240s.tif]

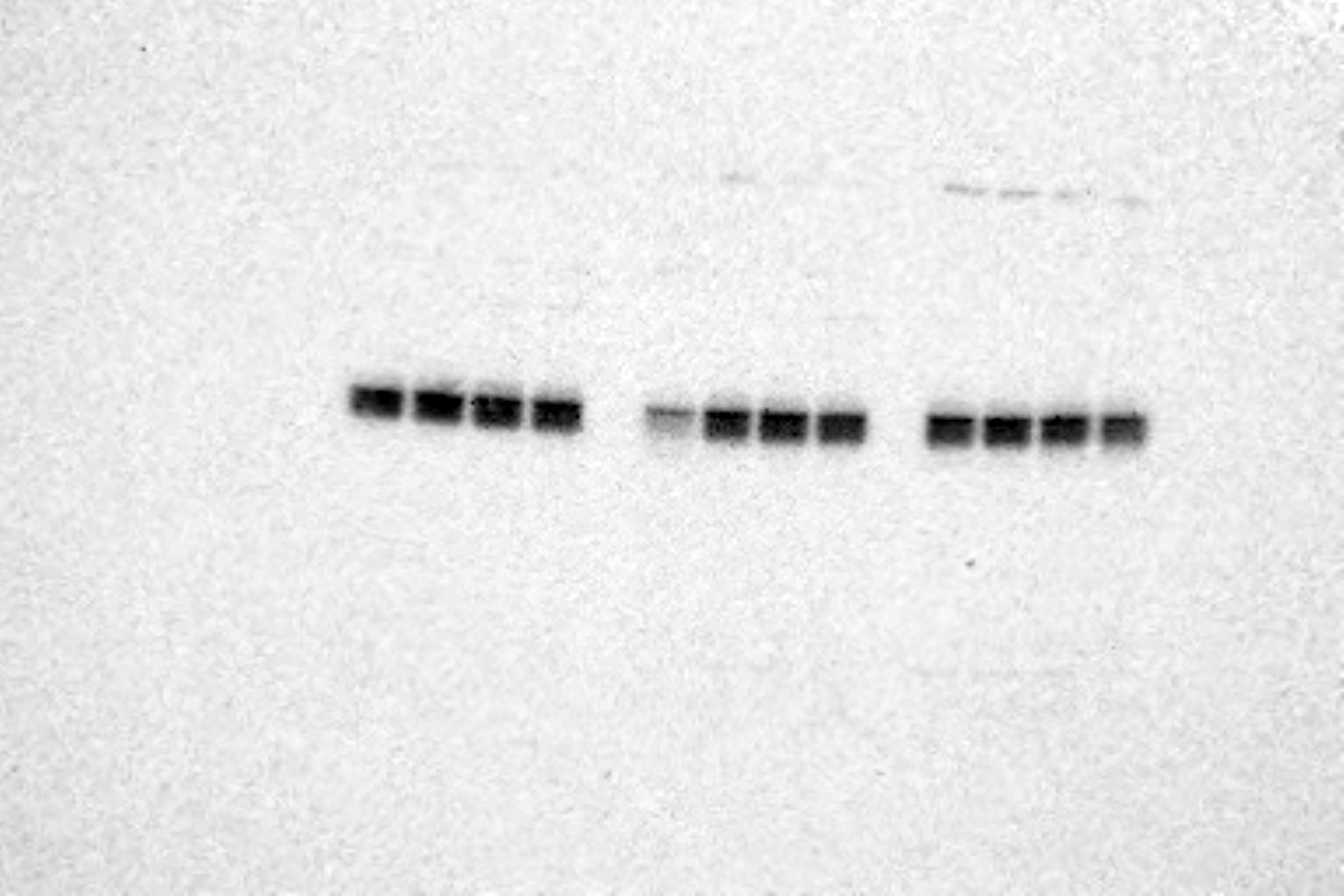

Supplement: Supplemental Information 1 — Actine, Catalase, Nrf-2. [file peerj-07-6565-s001.zip › nrf2-super-150s.tif]
